# Influence of Diet on the Bioavailability of Active Components from *Zingiber officinale* Using an In Vitro Digestion Model

**DOI:** 10.3390/foods12213897

**Published:** 2023-10-24

**Authors:** Justyna Zagórska, Karolina Pietrzak, Wirginia Kukula-Koch, Marcin Czop, Julia Laszuk, Wojciech Koch

**Affiliations:** 1 Department of Food and Nutrition, Medical University of Lublin, 4a Chodzki Str., 20-093 Lublin, Poland; justyna.zagorska@umlub.pl (J.Z.); karolina.pietrzak@umlub.pl (K.P.); la.julia@o2.pl (J.L.); 2 Department of Pharmacognosy with Medical Plants Garden, Medical University of Lublin, 1 Chodzki Str., 20-093 Lublin, Poland; virginia.kukula@gmail.com; 3 Department of Clinical Genetics, Medical University of Lublin, 11 Radziwiłłowska Str., 20-080 Lublin, Poland; marcin.czop@umlub.pl

**Keywords:** ginger, gingerol, shogaol, LC-MS

## Abstract

Ginger (*Zingiber officinale* Rosc.) is a plant known all over the world that is used as a spice and as an ingredient in drinks, dietary supplements, and cosmetics. The growing availability of its fresh rhizomes makes it even more likely to be used in the diet, mainly due to its beneficial health properties and high content of polyphenols (gingerols and shogaols). The main goal and motivation of the authors was to assess the bioavailability of active substances contained in the extract from ginger rhizomes in the presence of various types of diets using the in vitro digestion method, enabling simulation of the processes occurring during the digestion and absorption of metabolites in the small intestine. For the qualitative and quantitative analyses, the HPLC-MS (High Performance Liquid Chromatography–Mass Spectrometry) and HPLC (High Performance Liquid Chromatography) techniques were used, respectively. Based on the obtained results, it was found that the best bioavailability of the selected ginger polyphenols (6-gingerol, 8-gingerdione, 8-shogaol, and 10-gingerdione) was estimated for a high-fiber diet, while the weakest results were obtained for standard and basic diets. In the case of the high-fiber diet, the bioavailability of the mentioned compounds was estimated as 33.3, 21.4, 6.73, and 21.0%, while for the basic diet, it was only 21.3, 5.3, 2.0, and 1.0%, respectively.

## 1. Introduction

Ginger (*Zingiber officinale* Roscoe) is one of the most important plants that has been used for centuries in Asian medicine [1]. It is commonly used in the form of a powder, extract, essential oil, or as an ingredient in various types of dietary supplements and teas. In addition, both its fresh and dried rhizomes have long been a popular spice used in the preparation of oriental dishes. The consumption of ginger has recently spread significantly as it became an additive to other meals and beverages [2,3]. Ginger is an example a plant that enriches the smell and taste of the prepared dishes, but that also has great therapeutic importance, which makes it a component of functional foods or nutraceuticals for the prevention and treatment of chronic diseases [4,5,6].

*Zingiber officinale* is a perennial herbaceous plant from the Zingiberaceae botanical family. As it grows in warm and humid climatic conditions (70–90%) at a temperature of about 19–28 °C, the cultivation of this plant is mainly concentrated around warm countries such as Nigeria, India, China, Nepal, Indonesia, and Bangladesh [7]. The final composition of the ginger rhizome depends on the method and place of its cultivation, drying, and storage conditions [8]. The more favorable elemental composition reaching large amounts of potassium and manganese with a lower content of sodium and heavy metals is confirmed for ginger grown on organic plantations [9]. Fresh ginger rhizome consists of 12.3% carbohydrates, 2.4% fiber, 2.3% protein, 0.9% fat, 1.2% minerals, and 80.9% moisture [10]. The content of various chemical compounds in the ginger rhizome, in particular those responsible for its specific smell, is also affected by mechanical processing, i.e., of peeling the raw material (where, based on sensory analysis, a more citrus-like and fresh smell was confirmed for the unpeeled rhizome, which was also characterized by a higher polyphenol content) [11,12].

Based on the widespread research conducted so far on the extremely rich chemical composition of the *Zingiber officinale* rhizome, it was concluded that it contains 194 structures of terpene compounds, 28 types of diarylheptanoid compounds, and a group of 85 isomers based on a scaffold of gingerol (including, among others, various gingerols, paradoles, gingerdiones, shogaols, or zingerines) [13]. In addition, the ginger rhizome is a source of many vitamins (among others: C, E, niacin, B_6_) and minerals (Ca, Mg, P, K, Na) [1]. The main active substances identified in the ginger rhizome are 6-gingerol and 6-shogaol, which are characterized by a wide spectrum of biological properties. Shogaols are products of gingerol dehydration and are present in large quantities in aged plants or in the processed rhizomes, influenced by an elevated temperature or that have been stored for a long time [14,15].

Ginger is as a widely available plant that can be used as a medicine and, in the case of milder disorders, it can replace hard-to-access or expensive synthetic drugs bearing adverse side effects [15]. The many beneficial properties of this plant include: the antioxidant [16,17,18,19,20], antimicrobial [21,22,23,24,25], antiparasitic [26,27], anti-inflammatory [28,29], antidiabetic [30,31], antiulcer [32], antiemetic [33,34,35,36,37], analgesic [38,39,40,41,42], and even anticancer [43,44,45,46] properties. Still, studies on the biological effects of ginger-based treatment are still being continued to better understand the impact of compounds present in the rhizome on the human body, including corresponding mechanisms and molecular pathways [47]. Also, the studies on ginger extract in terms of its anti-aging activity are interesting. The plant’s activity is related to the senomorphic activity and anti-inflammatory effects. Researchers consider age-related diseases (ARDs) to be caused by long-term sterile inflammation, called inflammaging, accompanied by a repeated activation of immune cells and the accumulation of senescent cells [48]. Recently, it has become more obvious how important it is to include antioxidant components in the daily food rations that can prevent oxidative stress and thus slow down cell degradation and aging of the body [49].

Studies on the effect of heat treatment and digestion on the antioxidant potential of ginger samples in comparison with fresh unprocessed rhizomes have already been described. The methods and conditions of the thermal treatment of ginger rhizomes have also had a significant impact on the quality of the dried product, the content of active substances, and the biological activity [3,50,51]. The implementation of UPLC/QTOF-MS (Ultra Performance Liquid Chromatography/Quadrupole Time of Flight-Mass Spectrometry) and UPLC-PDA (Ultra Performance Liquid Chromatography–Photodiode Array Detection) methodologies provided sufficient evidence to state that the content of phenolic compounds in the extracts decreases in the following order: dried ginger, stir-fried ginger, fresh ginger, and carbonized ginger [52]. However, studies on the influence of thermal treatment on the composition of the ginger rhizome are inconclusive and it is hard to unequivocally state which method is the most preferable to retain or increase its biological properties. Nevertheless, it seems that cooking and microwaving have the most destructive effect on the total polyphenol content, while frying and blanching are the most preferred processing techniques [3,50]. It is worth noting that digestion has a significant impact on the composition and biological properties of ginger. It was found that this process negatively affects the oxidative capacity of ginger. The smallest decrease was calculated for oral digestion in the presence of saliva. The obtained results confirmed a protective role of saliva against pepsin and hydrochloric acid towards compounds with antioxidant properties [53].

As mentioned above, ginger rhizomes are characterized by a high content of metabolites with a wide spectrum of activity, including strong antioxidant activity. The consumption of spices and herbs, including ginger rhizomes, can be an important way to deliver natural antioxidants with pharmacological significance [54]. For this purpose, the used extracts must be standardized and show an adequate bioavailability, i.e., be released during digestion and absorbed into the bloodstream in a certain amount [55]. 

Bioavailability can be defined as the extent and rate at which active substances are absorbed effectively into a living organism [56]. The terms *bioaccessibility* and *bioavailability* are alternatively used in the scientific literature, but for the studies in which the dialysis tubes are used to filtrate a digested fraction, the term bioavailability is the most frequently used [57,58,59,60]. Even if, in general, in vitro methods do not reflect probably enough the typical rate of metabolites’ entry into the circulation, in our opinion, it seems that methods based on the application of cellulose membranes resemble natural conditions in the gastrointestinal tract to a greater extent, which is why we decided to follow this methodology and assess the bioaccessible fraction. This approach is a popular method to evaluate the influence of digestion on various nutrients or plants’ constituents. Therefore, the aim of this study was to assess the bioavailability of the main active compounds present in the ginger rhizome under the influence of various types of diet using the method of in vitro digestion (gastric and intestinal) and dialysis membranes. Thanks to the application of high-resolution mass spectrometry (HPLC-MS technique), phenolic compounds such as gingerols and shogaols were identified in the matrix, while when using HPLC, their content in the extract and digestion fractions was quantified to estimate their bioavailability. Our research sheds new light on the problem related to the bioavailability of active substances present in *Zingiber officinale* Rosc. Both ginger and products containing its preparations are currently very popular. The innovation of our research relies not only on the evaluation what the impact of the digestion process on the absorption of active compounds from this plant is, but also how the bioavailability of these compounds is influenced by the complex food matrix. While reviewing the scientific literature, we did not come across such broadly focused studies that evaluated the impact of various types of diets on the bioavailability of *Zingiber officinale* Rosc. components. Our research highlights how important the composition of our diet is (both in terms of quality and quantity) and provides valuable tips on which products to choose and combine with ginger polyphenols to ensure the best possible bioavailability of these compounds, which will translate into their stronger health-promoting effects. Our experiment may initiate greater attention not only to the impact of digestion, but also the composition of the diet on the bioavailability of plant substances with potentially health-promoting properties taken in the form of drugs, dietary supplements, or functional foods.

## 2. Materials and Methods

### 2.1. Plant Material

*Zingiber officinale* Rosc. rhizome was purchased in Lublin, Poland in a local market. Fresh ginger rhizome (unpeeled) was cut into small pieces and evenly spread on a tray, which was then placed in a laboratory dryer and left there for 60 h at 37 °C. After this time, the plant material was removed from the oven, left to cool down at a room temperature, and weighed. Less than 51 g of dried rhizome was obtained after drying from 306 g of fresh material.

### 2.2. Chemicals and Reagents

To obtain ginger extract, 96% ethanol (Avantor Chemicals, Gliwice, Poland) was used. For the sake of this study, ultrapure water (resistance 18.2 MΩcm) obtained using an Ultrapure Millipore Direct-Q-R 3UV (Millipore, Bedford, MA, USA) was obtained. The following reagents were used to carry out the in vitro digestion process: NaCl, HCl, NaHCO_3_ (Avantor Performance Materials Poland S. A., Gliwice, Poland), pepsin (77160, powder, slightly beige, ≥500 U/mg), pancreatin from porcine pancreas (P7545-25G), and bile extract porcine (B8631-100g, powder) (Sigma-Aldrich, St. Louis, MO, USA). As a reference compound for quantitative analysis, a standard of [6]-gingerol (≥98% HPLC) purchased from Sigma-Aldrich was used. Water and acetonitrile of HPLC grade were obtained from ChemSolve (Lodz, Poland) and JT Baker (Phillipsburg, NJ, USA). HPLC-MS analysis was performed with water, acetonitrile, and formic acid for HPLC-MS analyses (Merck, Darmstadt, Germany). 

### 2.3. Preparation of Zingiber Officinale Extract

Ginger extract was performed using a modified protocol, which was described previously [9]. Briefly, the dried ginger rhizome was divided into five Erlenmeyer flasks (each contained 10 g of ginger) and mixed with 50 mL of 96% ethanol. The flasks were placed in an ultrasonic bath Emmi-55HC-Q (EMAG Technologies, Mörfelden-Walldorf, Germany) and extracted for 30 min (temperature 30 °C, power 100%). After completion of the first stage of extraction, the liquid was filtered through tissue paper filter type 388 (Munktell, Falun, Sweden) into a large flask, and the residue in the flasks was mixed with 50 mL of 96% ethanol again and placed in an ultrasonic bath for another 30 min. After the second extraction process, the contents of all Erlenmayer flasks were filtered and combined with the extract from the first extraction step. Double ultrasound-assisted extraction was used to increase the efficiency of the process.

**Figure 1:**
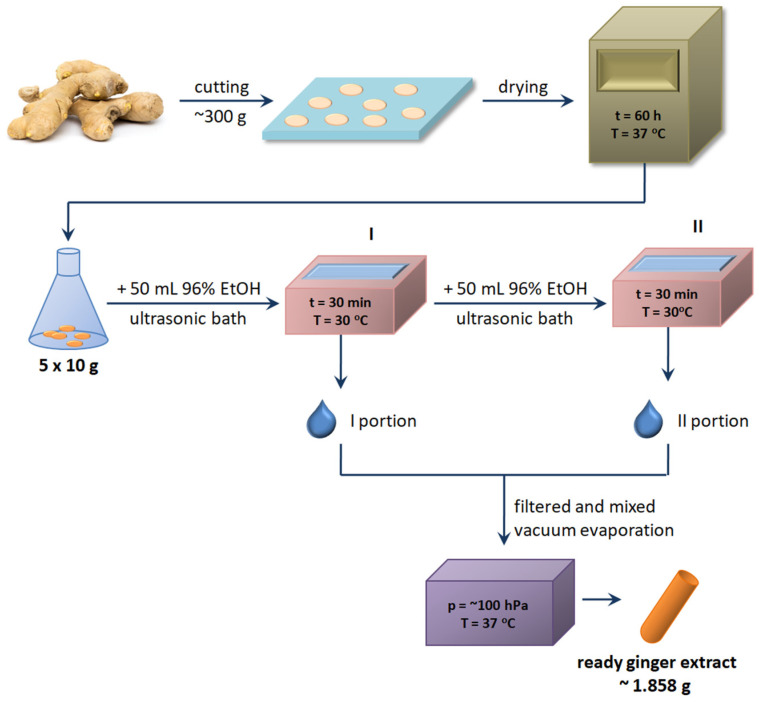
Experimental design of ginger extract preparation.

The solvent (ethanol) from the obtained raw fluid extract was evaporated using a vacuum evaporator Ingos RVO400 (INGOS, Prague, Czech Republic) under reduced pressure (~100 hPa) and a relatively low temperature (<37 °C) to prevent the decomposition of active substances present in the extract. The centrifuge, Eppendorff concentrator plus (Eppendorf, Warsaw, Poland), was used for the subsequent solvent evaporation step. Ultimately, 1.858 g of concentrated extract was obtained. The entire process of obtaining ginger extract is presented in Figure 1.

### 2.4. Diets Composition

Three types of diets were used for bioavailability research: a high-fiber diet, basic diet, and standard diet. Detailed information on the development and preparation of the diets has been previously described [61]. The exact composition of the diets and their nutritional values were summarized in the Supplementary Materials (Tables S1 and S2).

### 2.5. In Vitro Digestion

#### 2.5.1. Control and Studied Samples

Two control samples were prepared, each in 3 repetitions. For the control samples, ultrapure water was added instead of the diets. Studied samples were performed with the addition of prepared diets. All other procedures and reagents (i.e., preparation of enzyme mixtures, subsequent stages of gastric and intestinal digestion, and also using dialysis membrane) were identical in both studied and control samples. The digestion and bioavailability testing protocol was previously developed and optimized [61]; however, in the current study, it was modified and adapted to investigate plant extracts. 

#### 2.5.2. Stomach Digestion

The samples were prepared by weighing 0.2 g of ginger extract into laboratory containers. Then, 2 mL of ultrapure water (control samples) or 2 g of homogenized diets (studied samples) was added. In the next step, 9.6 mL of a mixture of 20 mg NaCl in 0.01 M HCl was added. The whole sample was mixed, and then the pH ~ 2 of the samples was adjusted using HCl acid (0.1 M). After obtaining an appropriate pH, 0.4 mL of pepsin solution in 0.1 M HCl was added. Closed containers were shaken in a water bath (Vibra, AJL Electronic, Krakow, Poland) for 2 h at 37 °C.

#### 2.5.3. Intestinal Digestion

After the gastric digestion, the pH of the residue was changed using NaHCO_3_ solution (1 M) to pH ~ 6.5, and then 5 mL of 4% pancreatin enzyme solution with the addition of bile salts (2.5% *w/v*) in 0.1 M NaHCO_3_ was added. The closed bottles were shaken again in a water bath for 2 h at 37 °C.

#### 2.5.4. Centrifugation

After the digestion process was finished, 5 mL of methanol was added to stop the enzymatic processes. Then, samples were centrifuged (10 min, 3000 rpm, Steinberg Systems centrifuge, Steinberg Systems, Berlin, Germany) and the volume was made up with ultrapure water to obtain 40 mL of each sample. 

#### 2.5.5. Analysis Using Dialysis Membranes

The obtained samples (V = 40 mL) were divided in half, half of which was filtered through a syringe filter and prepared for HPLC analysis. The other half of the solution (V = 20 mL) was put in a dialysis membrane with the Molecular weight cut-off (MWCO) of 2 kDa (Dialysis Tubing, Benzylated, Sigma-Aldrich, St. Louis, MO, USA) and tightly closed using laboratory clips (Sigma-Aldrich, St. Louis, MO, USA). The membranes were previously prepared by soaking in 0.1 M HCl for 12 h, and then rinsed with ultrapure water. A filled dialysis tube was put inside a laboratory container containing 200 mL of the ultrapure water. The closed containers were shaken again in a water bath for 2 h at 37 °C. Since during the filtration through the membranes the concentration equilibrium was established and the fluid volume inside the membrane changed, the fluid volumes were measured after the filtration process was completed, which was considered in further bioavailability calculations. The fluid inside the membrane that remained after this process was filtered through a syringe filter (Cronus Filters, Gloucester, UK, pores Ø 0.22 µm) for further analysis.

**Figure 2:**
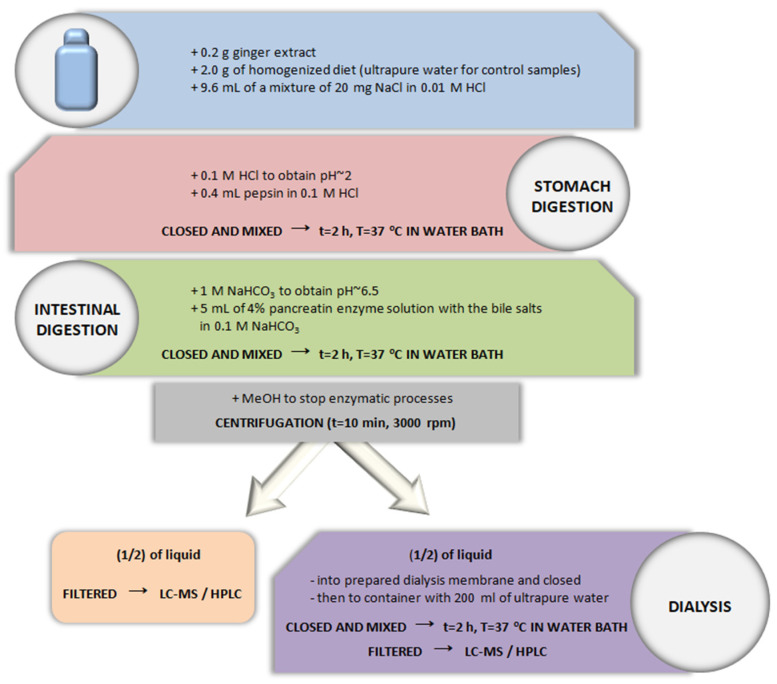
Digestion procedure in in vitro environment.

A scheme showing all stages of in vitro digestion is shown in Figure 2.

### 2.6. Qualitative Analysis Using LC-MS Method

The HPLC-ESI-QTOF-MS (High Performance Liquid Chromatography-Electrospray Ionization-Quadrupole Time of Flight-Mass Spectrometry) platform of Agilent Technologies company (Santa Clara, CA, USA) was used to analyze *Zingiber officinale* extract. A HPLC chromatograph (1200 series) with the Zorbax Eclipse Plus RP-18 column (150 mm × 2.1 mm; d_p_ = 3.5 µm) was equipped with a degasser (G1322A), binary pump (G1312C), autosampler (G1329B), photodiode detector—DAD (G1315D), and a mass spectrometer (G6530B). Mass Hunter Workstation software (Agilent Technologies, Santa Clara, CA, USA, B.10.00 version) was used to acquire the MS spectra and process the data.

Ginger extract was dissolved in methanol (at a concentration of 10 mg/mL) and then it was filtered through a syringe filter (Cronus Filters, pores Ø 0.22 µm) prior to the chromatographic determinations. LC-MS fingerprinting of samples was used to identify the compounds. During the analysis, the gradient elution mode was used (45 min; injection volume 5 µL) for mobile phase composition of eluent A: water with 0.1% HCOOH addition, *v*/*v*; eluent B: ACN with 0.1% HCOOH addition, *v*/*v* (flow rate 0.2 mL/min) changing over time (solvent A/solvent B): 10 min—55%/45%, 15 min—35%/65%, 25 min—20%/80%, 33 min—5%/95%, and 34 min—70%/30%. The operating range of the DAD detector was 190–400 nm. Collected spectra were scanned in m/z range 100–1700 Da in negative ionization mode. The other analysis parameters were, respectively: 275 °C and 325 °C for carrier and shielding gas, 12 L/min—gas flow velocity, 3000, 110, and 65 V–voltage of the capillary, slicer, and skimmer, 35 psig—nebulizer pressure, 10 and 20 V—collision energy. Two of the most intense signals visible in the TIC spectrum were automatically fragmented to obtain MS/MS spectra. After collecting two spectra for a given *m*/*z* value, selected signals were excluded from further fragmentation for 0.3 min.

### 2.7. Quantitative Analysis Using HPLC Method

For quantitative determinations, samples were analyzed using a chromatograph Prominence-i LC-2030 3D with DAD detection (Shimadzu, Japan) and column Zorbax Eclipse Plus RP-18 (150 mm × 2.1 mm; d_p_ = 3.5 µm) (Agilent, USA). All samples analyzed by HPLC were prepared by filtration through a syringe filter (Cronus Filters, Ø pores 0.22 µm). Solvent gradient elution mode was used: eluent A: ultrapure water; eluent B: acetonitrile; 0.1% of formic acid was added to both phases. The settings of the chromatograph were as follows: a thermostat temperature of 25.0 °C, flow rate of 0.2 mL/min, injection volume of 10 µL, UV detection range of 190–500 nm, and DAD detection wavelength of 290 nm. A single analysis lasted 35 min and the mobile phase composition (solvent A/solvent B) was as follows: 0 min—99%/1%, 3 min—70%/30%, 13 min—55%/45%, 18 min—35%/65%, 28 min—20%/80%, 30 min—5%/95%, and 33 min—99%/1%. The 6-gingerol standard solution (1.0 mg/mL in methanol) was used as an external standard for quantitative determinations. The calibration curve was plotted for the standard and the following equation was obtained: y = 5445203.358x + 55320.673 (R^2^ = 0.999). The linearity range covered the determined concentration of compounds in the samples and was calculated as 30–300 mg/g of the extract. The recovery of 6-gingerol was 95% and the repeatability of the quantitative determinations was higher than 97%. The limit of detection (LOD) value expressed as signal-to-noise (S/N) times 3 for the standard in the prepared method was calculated to be 61.9 µg/g (*n* = 5), whereas the limit of quantification (LOQ) calculated as S/N times 10 was 204.3 µg/g of the extract (*n* = 5). Because all identified compounds in the extract have similar structures and behave the same in the following chromatographic conditions, their concentrations were calculated based on the calibration curve plotted for 6-gingerol.

### 2.8. Statistical Analysis

Statistical analysis was performed using Statistica 13.3 (StatSoft, Kraków, Poland). Data were presented as mean with SD. The Shapiro–Wilk test was used to evaluate the normality of the data distribution. One-way ANOVA with Tukey post-hoc test was used. Results were considered statistically significant when *p* ≤ 0.05.

## 3. Results

### 3.1. HPLC-MS Fingerprinting of Zingiber Officinale Extract

**Figure 3:**
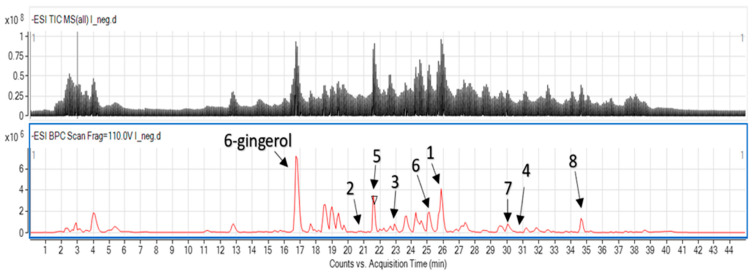
LC-MS chromatogram of ginger extract: 10-gingerdione (1); 8-gingerol (2); 8-shogaol (3); 12-gingerdione (4); 8-gingerdione (5); 6-dehydrogingerdione (6); 8-dehydrogingerdione (7); 10-dehydrogingerdione (8).

The applied gradient settings provided sufficient conditions for the separation of metabolites present in the total extract. The following compounds were identified in the sample as the major constituents: 6-gingerol, 10-gingerdione, 8-gingerol, 8-shogaol, 12-gingerdione, 8-gingerdione, 6-dehydrogingerdione, 8-dehydrogingerdione, 10-dehydrogingerdione. In the Table 1, the MS parameters received from the instrument are presented, which enabled the identification of these phenolic constituents in the mixture. Figure 3 shows the obtained chromatogram with identified compounds in the studied extract.

### 3.2. HPLC Determinations

**Figure 4:**
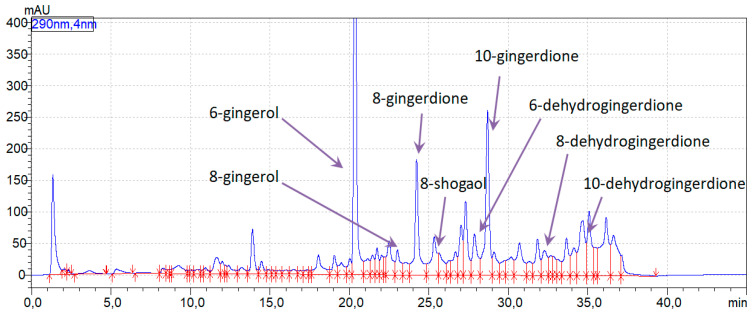
HPLC chromatogram of ginger extract with identified active compounds (extract concentration 10 mg/mL, injection volume 5 µL).

In the Figure 4, the chromatogram obtained for the studied ginger extract is presented. The chromatographic peaks of the compounds identified by LC-MS were appropriately described. HPLC determinations were performed for all samples, including the control and studied samples (in both compartments, before and after filtration through cellulose membrane). Quantitative determinations and bioavailability were calculated for four active compounds, which were presented in the extract in the highest amounts: 6-gingerol, 8-gingerdione, 8-shogaol, and 10-gingerdione. In Table 2, the values of the content of active substances in the ginger extract calculated based on the HPLC analysis are presented.

### 3.3. Bioavability Results

The bioavailability parameter was calculated taking into account the content of the studied active substances remaining in the mixture after digestion (gastric and intestinal) and the fraction obtained after filtration through a dialysis membrane in relation to the empirically determined content in the unprocessed ginger extract, according to the following formula:

$$\%\;Bioavability=\left[\frac{Ia-Ib}{Ic}\right]\times 100\%$$

where: *I_a_*—content after digestion, *I_b_*—content after digestion and filtration, and *I_c_*—initial content in the extract (before digestion).

The calculated results of the bioavailability of the selected active compounds are presented in Table 3. The greatest difference in bioavailability was shown for the 8-gingerdione and 10-gingerdione. In each of these cases, statistically significant differences were shown between the control samples, the high-fiber diet, the basic diet, and the standard diet. For 6-gingerol, significantly lower bioavailability was shown in the presence of the basic diet compared to the others. For 8-shogaol, the highest bioavailability was shown in the control samples and the lowest in the basic diet.

Table 4 presents a comparison of the content of compounds present in the fresh ginger extract and after the digestion process, depending on the type of diet. After digestion, the concentration of all studied substances was significantly reduced compared to the extract. In the case of 10-gingerdione, statistically significant differences were shown between all studied samples. In the case of 6-gingerol, after digestion, the highest reduction was observed in samples processed using a basic diet. In the case of 8-shogaol, the digestion process caused a significant reduction in comparison to the fresh extract; however, the differences between particular diets were not statistically significant. In the case of 8-gingerdione, the digestion process caused a significant reduction for all types of diet.

## 4. Discussion

Polyphenols (gingerols and shogaols) constitute a very important group of active compounds synthesized in ginger rhizomes. Extracts of many plants with health-promoting properties, including ginger, are widely studied from various aspects (especially regarding antioxidant activity [49], biological activity [47], or immunomodulatory effects [62]). However, in the case of the bioavailability of active substances after the in vitro digestion process, it is still not fully known and requires extensive research, especially due to the great popularity of ginger and products containing its preparations. Ginger and its extracts have been and still are studied in many ways, often also in clinical trials on patients. 

To reach the level of clinical trials, in vitro studies are necessary, as they can somehow direct the expensive and long-term in vivo studies that are performed on laboratory animals or humans. In terms of studies on bioavailability, in vitro tests play an important role. During the simulation of the digestion processes taking place in a living organism, the conditions (including the pH of the environment and the presence of appropriate enzymes or the use of membranes) can be appropriately adjusted to reflect in vivo digestion as accurately as possible [55,63]. 

In the literature, there are many publications on the composition of different plant species, including ginger, and data on the biological properties of extracts. However, there are very few studies on the bioavailability of plant active substances, either in vitro or in vivo [64,65,66]. The estimation of the degree of bioavailability of active components creates many new possibilities for the usage of ginger and an improvement of the existing ones in the pharmaceutical and food industries (including the design and production of new drugs, dietary supplements, and functional foods) [67]. An equally important aspect related to the bioavailability is the possibility of a more accurate determination of the ginger dose or single active components in pharmaceuticals to induce a specific therapeutic effect, considering that only some parts of active compounds are transferred into the circulation. What is more, establishing the right type of diet (both qualitative and quantitative parameters) when consuming ginger or its preparations is crucial in order to minimize disturbances in the absorption of its active components from the gastrointestinal tract because compounds presented in food (including proteins, carbohydrates, or lipids) can have a significant impact on the activity of phenolic compounds as a result of their various interactions [68].

To the best of our knowledge, our study is the first in which the bioavailability of various active compounds from ginger was determined using an in vitro model with cellulose membranes in the presence of various types of diet. Therefore, it is hard to discuss the obtained results for ginger, as there are limited studies on this topic. We found only two studies that evaluated the effect of simulated digestion in the gastrointestinal tract on the active compounds in ginger. Wang et al. studied the content of ginger polyphenols in the simulated digestion process, as well as their prebiotic function in relation to intestinal microflora and the in vitro fermentation process. Based on the obtained results, the authors found that about 85% of polyphenols were still detectable after the in vitro digestion process, and the presence of ginger extract had a positive effect on the growth of some populations of desirable bacteria and modulated the structure of microflora, which may confirm the potential use of 6-gingerol as a prebiotic agent [69]. The stability of ginger metabolites in the conditions of gastrointestinal digestion (including the use of artificial digestive juices such as saliva, gastric juice, duodenal juice, and bile) was also investigated by Annuziato et al. The authors identified a number of chemical compounds present in the ginger rhizome and conducted a comparative analysis before and after digestion using the LC/ESI-MS/MS method. Based on the obtained results, it was found that there are no significant differences in the amount of bioactive compounds present in ginger before and after the process of gastrointestinal digestion, and it is possible to absorb them from the small intestine in an unchanged form [70].

Moreover, there are also not many studies related to the bioavailability of plant-derived natural products found in the scientific literature. The change in the bioavailability of polyphenols was investigated, among others, by Sęczyk et al., using in vitro digestion and hydrothermal treatment in the presence of food matrices, which, however, consisted only of various types of flour, without the addition of other food products. It was found that most of the activities had a negative effect, and the potential bioavailability of phenolic compounds depended on many factors (type of compound, food matrix, and method of processing) [71]. Cebeci et al. studied the effect of a matrix containing blueberries and oatmeal with the addition of various types of milk (whole and skimmed) on the bioavailability of polyphenols. The authors found that a meal consisting of the studied products also with the addition of milk did not significantly affect the change in the bioacccessible fraction from the small intestine. At the same time, the authors observed that the addition of milk had an inhibitory effect on the total content of polyphenols, which was probably the result of interactions between polyphenols and milk proteins [72]. Further in vitro studies were described by Pešić et al. The authors’ aim was to investigate the effect of the food matrix on grape extract digestion (composition of polyphenols, their content, bioaccessibility, and antioxidant activity during digestion). It was found that the presence of a food matrix containing significant amounts of phenolic acids and flavonols resulted in a significant increase in the total recovery of polyphenols after digestion compared to the sample containing no food matrix. Moreover, the increase in antioxidant activity was higher in the case of grape skin extracts than in the case of grape seed extracts. In this case, according to the authors, their research has shown that the addition of grape extracts to cereal and meat products had a positive effect on health-promoting properties of the diet. The research has confirmed that providing the organism with active substances in the form of enriched products is a much better solution, rather than in the form of artificial pills and supplements. Moreover, it was concluded that it is better to consume products rich in polyphenols during a meal than on an empty stomach [73]. In turn, Dufour et al., for the first time, investigated polyphenol bioaccessibility in an in vivo environment (minipigs) in the presence of a food matrix with the addition of fruit and vegetables (apples, plums, and artichokes) or phenolic extract. Importantly, the dose of phenolic extract (22.8 g) contained the same number of polyphenols as the fruit and vegetables portion. The authors estimated polyphenol bioaccessibility in stomach at 1.5 and 3.1% after the addition of fruits and vegetables or extract ingestion, respectively. It was found that in the case of protein digestion, the presence of polyphenols from fruits and vegetables had no negative effect, while in the case of the addition of phenolic extract, it had. These authors have also observed an important interaction between polyphenols and proteins. It was also concluded that due to the reduction in the speed and efficiency of protein digestion in the presence of polyphenol extract, people with a low protein content in their diet should pay special attention to products containing such extracts (dietary supplements, medicines, functional foods) [74].

In the present study, the most important parameter that has been determined is bioavailability, which is the basis for understanding how phenolic substances behave in the gastric and intestinal environment and what percentage of active ginger compounds is able to be absorbed from the intestines and show potential pro-health activity. Many various factors have an influence on the bioavailability of polyphenols, including the properties and sizes of the molecules, their chemical states, interactions with other substances, and the food matrix during the digestion process taking place at variable pH conditions depending on its stage. During stomach digestion (low pH), flavonoid oligomers are decomposed into smaller units to undergo reactions in the small intestine (higher pH), including, among others, methylation, deglycosylation, hydroxylation, and sulfonation of flavonoids [75]. Therefore, studying the bioavailability of compounds present in the ginger rhizome in the presence of the food matrix is complicated, but also important in order to be able to properly determine the dose of the extract necessary to achieve the desired therapeutic effect. Estimating the impact of the type of the diet that is consumed simultaneously with ginger or its preparations enables us also to adjust its composition, both qualitative and quantitative, to obtain optimal results. Being aware that the simulation of in vitro digestion does not accurately reflect in vivo conditions, as part of the conducted research, efforts were made to simulate this process in the best possible way, especially considering the appropriate gastric and intestinal environment (pH, bile salts, enzymes: pepsin, pancreatin), maintaining a temperature similar to the temperature of the human body (~36–37 °C), and digestion time (2 h stages). It has been shown that the digestion process itself reduces the amount of ingested active compounds that can potentially be absorbed in the small intestine, which may be caused by binding reactions between phenolics and mixtures of pancreatin/bile salt and can be related to the above-mentioned factors affecting this process [76]. In the present work, it has been shown that the digestion process itself significantly reduces the initial content of the studied substances in the range of 55–99%, depending on the type of compound and the diet with which it was mixed. Among the studied compounds, 6-gingerol was characterized by the highest bioavailability in each diet, and 8-shoagol, by the lowest. However, 8-gingerdione and 10-gingerdione, depending on the diet, had intermediate values and changeable order. It was observed that the basic diet had the most negative effect on the bioavailability of all studied compounds. The calculated reduction of their concentration after digestion was very high—71, 94, 98, and 99%, for 6-gingerol, 8-gingerdione, 8-shogaol, and 10-gingerdione, respectively—which means that only a small percentage of them may be absorbed in the small intestine and passed into the blood. In the case of a standard diet, these values were: 56, 80, 94, and 86%, respectively, while the lowest influence of the diet matrix on the concentration of most polyphenols after digestion process was observed for the high-fiber diet, and these values were 55, 77, 93, and 78%, for 6-gingerol, 8-gingerdione, 8-shogaol, and 10-gingerdione, respectively. The high-fiber diet was characterized not only by the highest content of carbohydrates (423.2 g) and fiber (50.3 g) among the diets used, but also by the highest content of vitamins (A, C, E) and minerals (Ca, K, Mg, Fe) (see Table S2 in Supplementary Materials). The basic diet was also characterized by high carbohydrate content (331 g), although the content of fiber (27.8 g), as well as vitamins and minerals, was much lower (27.8 g). The standard diet was characterized by a moderate content of basic nutrients and vitamins and minerals. 

The obtained results can be related to the real processes occurring in the human body and can analyze how active compounds from *Zingiber officinale* behave during digestion in various sections of the digestive tract, although they also have some limitations. First of all, the results of the present study were obtained in a model of simulated in vitro digestion using cellulose membranes, which may cause the obtained results to be different from animal or human studies, as mentioned above. Moreover, the use of ginger or its extracts is very individual, as these products are used at different times of the day, with different meals, and with different degrees of stomach filling. Therefore, in this study, typical diets used in nutrition were mapped to show the prevailing trends in how the composition of the food ration can affect the bioavailability of various active compounds from the ginger rhizome. The strength of the current study is that conclusions were based on analytical determinations using very sensitive, precise methods (including compound identification by means of mass spectrometry) and thorough statistical analysis. In the current study, the direct influence of a high protein content on the reduction of phenolics from the ginger rhizome was not observed; however, all diets used in the study were characterized by a high protein content, which may explain why the digestion process itself significantly reduced the initial concentration of all studied compounds. As was previously mentioned, significant interactions between proteins and phenolics that resulted in the decreased bioavailability of these compounds have been observed [65,67]. Pešić et al. [66] revealed that the bioavailability of phenolics from grapes was higher in the presence of a food matrix in comparison to an empty stomach. In the current study, the obtained results were totally different, as the bioavailability for all studied compounds was the highest for control samples (without any diet), which represented an empty stomach. These differences may be due to the different structure of phenolics from the ginger rhizome in comparison to grapes and also because of the different model used during bioavailability investigations. 

## 5. Conclusions

In the current study, the bioavailability of four main active compounds in the *Zingiber officinale* rhizome extract was determined: 6-gingerol (the highest content), 10-gingerdione, 8-gingerdione, and 8-shoagol. It has been shown that the presence of a food matrix reduces the bioavailability of the studied active compounds, while the degree of the effect depends on the type of diet. In the case of each diet, among the studied compounds, 6-gingerol was characterized by the highest bioavailability, followed by 8-gingerdione, 10-gingerdione, and 8-shoagol. In general, it can be concluded that the bioavailability of the investigated compounds decreased in the following order: high-residue diet (diet with high fiber content) >> standard diet >> basic diet. It was found that the digestion process itself significantly reduced the amount of active substances compared to the initial content in the analyzed extract. Based on the obtained results, it can be concluded that the type of food matrix has a significant impact on the bioavailability of polyphenols present in the ginger rhizome and that a balanced diet promotes their positive interactions with nutrients, which can act as carriers and protectors against oxidative degradation. The high-fiber diet was characterized by the highest content of vitamins with antioxidant properties, which could protect polyphenols from oxidative processes. It may also be assumed that the smallest decrease in the bioavailability for a high-fiber diet (highest in calories, highest in vitamins and minerals) is likely due to the high fiber and vitamin C content. Moreover, we noticed that as the amount of proteins and fat in the diet decreased, the bioavailability of the studied compounds also decreased. The high-fiber diet was characterized by the highest protein and fat content, which resulted in the best bioavailability of ginger’s active substances. The relation we noticed between the bioavailability of the determined phenolic compounds and the content of minerals in the diets used is surprising. The basic diet was characterized by a much lower content of each mineral (calcium, sodium, potassium, magnesium, and iron) compared to the other diets, and at the same time, the combination of ginger extract with this diet resulted in the lowest bioavailability of the determined active substances. It is worth emphasizing that our study did not focus on the impact of individual nutrients on bioavailability, but the results we obtained can be considered as preliminary research that will be the basis for future experiments on the impact of individual components of food products on the bioavailability of active substances obtained from *Zingiber officinale* Rosc. and other plants. This is an important issue that requires further research because the effectiveness of the beneficial effects of plant ingredients on the human body depends on their bioavailability, which is influenced by the digestion process and its complex interactions with food components, not only on their concentration in the raw plant material. The results of our research can be used in practice during composing a diet that will ensure the best absorption of ginger’s active substances (from the plant, dietary supplements, functional foods, or pharmaceuticals), which, in turn, will result in an increase in the health-promoting effects of these compounds.

## Figures and Tables

**Table 1:** The HPLC-MS parameters recorded in the negative ionization mode for the analyzed ethanolic extract from *Zingiber officinale* rhizomes.

No.	Ion (+/−)	Retention Time	Molecular Formula	m/z Calculated	m/z Experimental	Delta (ppm)	RDB	MS/MS Fragments	Proposed Compound
1	−	25.8	C_21_H_32_O_4_	347.2228	347.2238	−2.92	6	191.0708 176.0472	10-gingerdione
2	−	20.7	C_19_H_30_O_4_	321.2071	321.2078	−2.07	5	259.2060 247.2092	8-gingerol
3	−	22.9	C_19_H_28_O_3_	303.1966	303.1973	−2.41	6	303.1969	8-shogaol
4	−	30.9	C_23_H_36_O_4_	375.2541	375.2549	−2.17	6	191.0696 176.0501	12-gingerdione
5	−	21.6	C_19_H_28_O_4_	319.1915	319.1920	−1.61	6	304.1685 191.0707	8-gingerdione
6	−	25.1	C_17_H_22_O_4_	289.1445	289.1445	0.11	7	274.1214 149.0607	6-dehydrogingerdione
7	−	30.0	C_19_H_26_O_4_	317.1758	317.1766	−2.41	7	167.1072 149.0608	8-dehydrogingerdione
8	−	34.6	C_21_H_30_O_4_	345.2071	345.2080	2.50	7	330.1835 175.0392	10-dehydrogingerdione
9	−	16.9	C_17_H_26_O_4_	291.1609	291.1605	2.45	6	191.0718 176.0480	6-gingerol

**Table 2:** Content [mg/g] of active substances in the ginger extract (calculated per 1 g of extract).

Compound	6-Gingerol	8-Gingerdione	8-Shogaol	10-Gingerdione
Mean value	162.0	60.0	44.1	76.7
SD	4.6	10.6	32.2	1.35

**Table 3:** Bioavailability [%] of active substances in the control (only water, no nutrients) and studied samples. The means not sharing the same letter in a column are significantly different at *p* ≤ 0.05.

	6-Gingerol	8-Gingerdione	8-Shogaol	10-Gingerdione
	Control sample			
Mean %B (n = 6)	35.46 ^a^	27.58 ^a^	13.89 ^a^	34.62 ^a^
SD	1.91	1.59	1.13	1.20
	High-fiber diet			
Mean %B (n = 3)	33.29 ^a^	21.42 ^b^	6.73 ^b^	20.99 ^b^
SD	1.40	0.0	0.13	0.27
	Basic diet			
Mean %B (n = 3)	21.32 ^b^	5.30 ^c^	2.0 ^c^	0.98 ^c^
SD	0.1	0.0	0.13	0.0
	Standard diet			
Mean %B (n = 3)	35.02 ^a^	17.78 ^d^	4.99 ^b^	13.69 ^d^
SD	0.03	0.09	0.0	0.2

**Table 4:** Content of active substances in studied samples. The means not sharing the same letter in a column are significantly different at *p* ≤ 0.05.

Content [mg/200 mg of Extract]	6-Gingerol	8-Gingerdione	8-Shogaol	10-Gingerdione
Extract	32.4 ± 0.92 ^a^	12.0 ± 2.12 ^a^	8.82 ± 6.43 ^a^	15.33 ± 0.27 ^a^
	After digestion			
Control sample (n = 6)	16.31 ± 0.06 ^b^	3.67 ± 0.05 ^b^	1.37 ± 0.04 ^b^	5.69 ± 0.10 ^b^
High-fiber diet (n = 3)	14.53 ± 0.46 ^c^	2.72 ± 0.0 ^bc^	0.65 ± 0.01 ^b^	3.33 ± 0.06 ^c^
Basic diet (n = 3)	9.31 ± 0.03 ^d^	0.7 ± 0.0 ^c^	0.21 ± 0.01 ^b^	0.18 ± 0.0 ^d^
Standard diet (n = 3)	14.41 ± 0.01 ^c^	2.31 ± 0.01 ^bc^	0.5 ± 0.0 ^b^	2.14 ± 0.0 ^e^

## Data Availability

Data are contained within the article or Supplementary Materials.

## Supplementary Materials

The following supporting information can be downloaded at: https://www.mdpi.com/article/10.3390/foods12213897/s1, Table S1: Composition of particular diets used in the study; Table S2: Nutritional values of diets used in the study.
