# Supplementary material for: Influence of Diet on the Bioavailability of Active Components from Zingiber officinale Using an In Vitro Digestion Model
Source: Foods. 2023 Oct 24;12(21):3897. doi: 10.3390/foods12213897 (PMC10648287; doi:10.3390/foods12213897)
Supplement: Supplementary file 1 [file foods-12-03897-s001.zip › foods-2671465-supplementary.pdf]

SUPPLEMENTARY FILE

Table S1. Composition of particular diets used in the study [61].

	High-fiber diet		Basic diet		Standard diet	
**Breakfast**	milk<br>oat flakes or others<br>wholemeal bread<br>cottage cheese<br>jam / 25 g	400 mL<br>50 g<br>80 g<br>100 g<br>25 g	cereal coffee + milk<br>light bread<br>butter<br>cottage cheese<br>(+ 10 g chives and 25 g cucumber)	250 mL<br>80 g<br>10 g<br>100 g	hot chocolate<br>mixed bread<br>ham sausages<br>mustard	250 mL<br>80 g<br>150 g<br>10 g
**Lunch**	tea<br>mixed bread<br>rennet cheese<br>ham<br>tomato<br>banana	250 mL<br>80 g<br>50 g<br>40 g<br>150 g<br>120 g	tea<br>mixed bread<br>chicken pate<br>apple	250 mL<br>60 g<br>120 g<br>150 g	coffee with milk<br>crispbread<br>gouda cheese	120 mL<br>20 g<br>50 g
**Dinner**	strawberry compote<br>red borscht (30g beetroot + 25 g vegetables: carrot, parsley, celery)<br>potatoes<br>pork chop<br>cooked vegetables (carrot + peas (1:1) with butter)<br>apple	200 mL<br>400 mL<br>300 g<br>150 g<br>300 g<br>150 g	strawberry compote with fruit pieces (30 g)<br>vegetable soup (100 g vegetables: carrot, celery, parsley, cauliflower)<br>potatoes<br>chicken cutlet<br>red cabbage salad	250 mL<br>400 mL<br>300 g<br>150 g<br>120 g	compote<br>coffee with milk<br>tomato soup with noodles<br>potatoes<br>grilled cod<br>sauerkraut salad<br>milk chocolate with nuts	220 mL<br>120 mL<br>400 mL<br>300 g<br>200 g<br>120 g<br>25 g
**Supper**	hot chocolate<br>mixed bread<br>butter<br>chicken ham<br>pepper	220 mL<br>80 g<br>10 g<br>80 g<br>150 g	tea with milk<br>meatballs in sauce<br>noodles<br>salad (carrot, apple, mayonnaise)<br>yeast cake with crumble	250 mL<br>100 g<br>120 g<br>100 g<br>50 g	tea<br>mixed bread<br>sausages<br>pickled cucumber	250 mL<br>60 g<br>80 g<br>70 g

Table S2. Nutritional values of diets used in the study [61].

	High-fiber diet	Basic diet	Standard diet
**Protein**	151 g (19% of energy)	104 g (16.4% of energy)	138.4 g (22.6% of energy)
**Fat**	120.3 g (24% of energy)	101 g (35.6% of energy)	114.4 g (42% of energy)
**Carbohydrates**	423.2 g (47% of energy)	331 g (47.1% of energy)	237.4 g (35.4% of energy)
**Fiber**	50.3 g	27.8 g	21.0 g
**Energy**	3186.6 kcal	2545 kcal	2451 kcal

**Vitamin A**	4.75 mg	2.83 mg	0.47 mg
**Vitamin C**	307.2 mg	76.6 mg	50.7 mg
**Vitamin E**	24.8 mg	15.7 mg	14.3 mg
**Calcium**	1618.5 mg	502 mg	1067 mg
**Sodium**	3410.8 mg	2207 mg	4053 mg
**Potassium**	6924.8 mg	4700 mg	5156 mg
**Magnesium**	689.3 mg	349 mg	497 mg
**Iron**	21.4 mg	13.6 mg	15 mg

The nutritional value of the diets was calculated using Dietetyk 2006 software (Jumar, Poland).
